# Functional conservation of a forebrain enhancer from the elephant shark (*Callorhinchus milii *) in zebrafish and mice

**DOI:** 10.1186/1471-2148-10-157

**Published:** 2010-05-26

**Authors:** Ryan B MacDonald, Mélanie Debiais-Thibaud, Kyle Martin, Luc Poitras, Boon-Hui Tay, Byrappa Venkatesh, Marc Ekker

**Affiliations:** 1 Center for Advanced Research in Environmental Genomics, Department of Biology, University of Ottawa, Ottawa, ON, K1N 6N5, Canada; 2 Institute of Molecular and Cell Biology, A*STAR, Biopolis, 138673, Singapore; 3 Department of Zoology, Oxford University, Oxford, UK

## Abstract

**Background:**

The phylogenetic position of the elephant shark (*Callorhinchus milii *) is particularly relevant to study the evolution of genes and gene regulation in vertebrates. Here we examine the evolution of *Dlx *homeobox gene regulation during vertebrate embryonic development with a particular focus on the forebrain. We first identified the elephant shark sequence orthologous to the URE2 *cis *-regulatory element of the mouse *Dlx1/Dlx2 *locus (herein named CmURE2). We then conducted a comparative study of the sequence and enhancer activity of CmURE2 with that of orthologous regulatory sequences from zebrafish and mouse.

**Results:**

The CmURE2 sequence shows a high percentage of identity with its mouse and zebrafish counterparts but is overall more similar to mouse URE2 (MmURE2) than to zebrafish URE2 (DrURE2). In transgenic zebrafish and mouse embryos, CmURE2 displayed enhancer activity in the forebrain that overlapped with that of DrURE2 and MmURE2. However, we detected notable differences in the activity of the three sequences in the diencephalon. Outside of the forebrain, CmURE2 shows enhancer activity in areas such as the pharyngeal arches and dorsal root ganglia where its' counterparts are also active.

**Conclusions:**

Our transgenic assays show that part of the URE2 enhancer activity is conserved throughout jawed vertebrates but also that new characteristics have evolved in the different groups. Our study demonstrates that the elephant shark is a useful outgroup to study the evolution of regulatory mechanisms in vertebrates and to address how changes in the sequence of *cis *-regulatory elements translate into changes in their regulatory activity.

## Background

Changes in gene expression patterns, via changes in *cis *-regulatory elements, or in the *trans *-acting factors binding to these elements, has contributed to the development of novel morphological structures during evolution [1]. The high degree of conservation in the coding region of genes necessary to establish the animal body plan has been extensively documented. The growing wealth of metazoan genome sequence data has also provided evidence for the conservation of sequences outside the coding regions of genes, the Conserved Non-coding Elements (CNEs) that tend to be located close to developmental genes [2,3]. However, whether the conservation of CNE sequence is necessary for any conservation of CNE regulatory activity remains debateable. Recent studies have identified regulatory sequences with very little sequence conservation that have the ability to activate transcription in highly similar tissues [4-7]. Furthermore, highly conserved regulatory sequences can drive transcription in highly divergent patterns [8-10]. Therefore, it remains challenging to predict regulatory activity based solely upon sequence similarity, or vice versa. Currently, a great effort has been made in systematically characterizing the CNEs in the mouse genome allowing for comparison with other model and non-model organisms [11].

*Dlx *homeobox genes of vertebrates are involved in the development of the forebrain, visceral arches, sensory organs, and limbs [12]. They are organized as three convergently transcribed bigene clusters, present in most jawed vertebrates: *Dlx1/Dlx2 *, *Dlx3/Dlx4 *and *Dlx5/Dlx6 *[13]. The three bigene clusters most likely originate from the duplications of an ancestral bigene cluster occurring as whole genome duplication events throughout vertebrate evolution [14]. A similar bigene arrangement of *Dlx *genes has been reported in the ascidian *Ciona intestinalis *[15], suggesting that the ancestral bigene existed prior to vertebrate radiation. The bigene organization of *Dlx *genes appears to be important for the concerted expression of the two genes within each cluster as *cis *-regulatory elements (CREs) have been identified in the relatively short (~3-15 kb) intergenic regions separating the two genes of each cluster [16,17].

Of the six *Dlx *genes found in tetrapod vertebrates, the *Dlx1/Dlx2 *and *Dlx5/Dlx6 *clusters are involved in forebrain development. We have previously reported three CREs from the intergenic regions of these two bigene clusters in mouse: I12b from the *Dlx1/Dlx2 *locus and I56i and I56ii from the *Dlx5/Dlx6 *locus [16,17]. However, CREs regulating *Dlx *expression can also be found outside the intergenic region and we reported one such CRE named Upstream regulatory element 2 (URE2), located approximately 12 kb upstream of the mouse *Dlx1 *gene [18]. We have previously shown some degree of conservation in the function of some *Dlx *CREs between mouse and zebrafish but it is not clear when these CREs and their associated regulatory mechanisms originated during vertebrate evolution [16,17].

As a cartilaginous fish, the elephant shark (*Callorhinchus milii *) occupies an interesting phylogenetic position as the sister group to bony fishes and tetrapods. Its relatively small genome of 910 Mbp and the availability of a low coverage genome (~1.4 ×) make this species a useful cartilaginous fish model to examine conservation of CREs [19]. Large scale comparison of the conserved non-coding regions between the elephant shark and the human and/or zebrafish genomes has revealed that more CNEs are shared between human and elephant shark than between human and zebrafish [19,20]. These data are consistent with the observation of greater conservation in gene synteny between human and elephant shark genomes than between human and zebrafish genomes [19]. This may seem counterintuitive given that mammals share a more recent common ancestor with teleost fishes than with cartilaginous fishes. However, this situation likely results from the 'fish-specific' whole genome duplication event that occurred before the teleost radiation and led to loss or modification of CNEs and a high level of genome re-organization in this group [19,21]. Detailed analysis of the conservation of CNEs associated with the *Hox *clusters in the elephant shark, human, and fugu yielded various hypotheses on the possible correlation between the level of sequence conservation of vertebrate CNEs and their functional variation [21].

Here, we report the identification of an elephant shark sequence orthologous to the conserved regulatory element URE2 associated with *Dlx1 *and *Dlx2 *genes. Sequence comparisons show a high level of conservation within gnathostomes, with higher similarity between elephant shark and mouse URE2 than between elephant shark and zebrafish URE2. We show that the enhancer activity of the elephant shark URE2 (CmURE2) in transgenic mouse and zebrafish is highly similar to that of its orthologous mouse and zebrafish counterparts in transgenic assays. In addition, CmURE2 shows more similarity in sequence and function to the orthologous mouse sequence than to the zebrafish sequence, in agreement with the hypothesis of additional genome and gene regulation remodelling due to the subsequent teleost specific genome duplication.

## Results

### Sequence and synteny conservation near the *Dlx1/Dlx2 *bigene cluster

**Figure 1:**
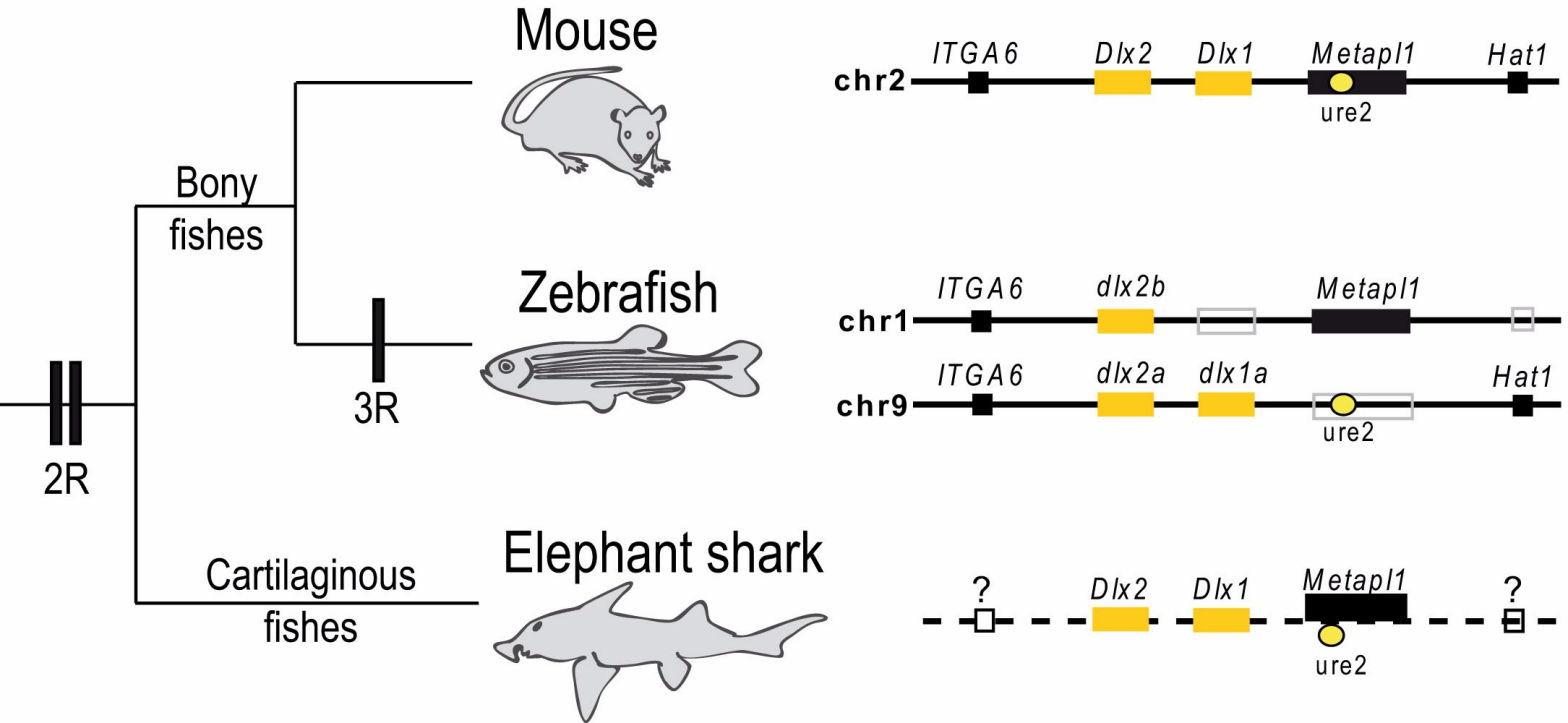
**The *Dlx1/Dlx2 *bigene cluster in vertebrate evolution**. Vertebrate phylogeny indicates the hypothesized position of the three genome duplication events (2R, 3R). The chromosomal neighbourhood of *Dlx1/Dlx2 *in mouse and zebrafish presents the relative position of flanking genes and of URE2. In the mouse, URE2 falls within intron 6 of the *Metapl1 *gene. Synteny information is not yet available for the elephant shark although the presence of the *Dlx1 *and *Dlx2 *genes on the same BAC clone as URE2 has been confirmed. Drawings are not to scale.

The *Dlx *genes of most tetrapod vertebrates described thus far are organized as three *Dlx *bigene clusters. As a result of the whole genome duplication event occurring in ancestral teleost fish, several other *dlx *genes have been identified in zebrafish; (i) an additional *dlx1a/dlx2a *bigene cluster located on chromosome 9, and (ii) a *dlx2 *-related gene, *dlx2b *, located on chromosome 1, which is not physically linked to a *dlx1 *-like gene [22,23]. Conserved synteny between the *dlx *-containing regions of zebrafish chromosomes 1 and 9 supports the hypothesis that *dlx1a/dlx2a *and *dlx2b *arose from the duplication of a large chromosomal region, followed by the loss of the *dlx1 *-like gene from the b cluster. Furthermore, the synteny is also conserved with a region of mouse chromosome 2 that contains the nearby genes *ITGA6 *, *Metapl1 *and *Hat1 *(Figure 1). The presence of CREs within these genomic regions may contribute to the conservation of these synteny blocks [24-26]. In the mouse, the MmURE2 CRE, previously reported to be involved in *Dlx1/Dlx2 *regulation and located 12 kb upstream of *Dlx1 *, falls within the sixth intron of the *Metapl1 *gene [18]. This conserved sequence is able to drive reporter expression in the mouse forebrain [11,18,27] . In zebrafish, DrURE2 is located in a similar position upstream of the *dlx1a *gene but the unique *metapl1 *ortholog is found in synteny with *dlx2b *on chromosome 1. No URE2-like sequence can be identified on zebrafish chromosome 1 (see synteny description at [28]) which rules out the possibility of a URE2 enhancer--like sequence acting on the *metapl1 *gene and suggests that the remaining *dlx2b *gene is not under the regulation of a URE2 sequence, except if the sequence has been highly remodelled after the duplication while still retaining its function. This, as well as the loss of other regulatory elements associated with the *dlx1a-dlx2a *bigene cluster [17], may account for the reduced domains of expression in comparison to *dlx2a *[29,30].

To investigate the corresponding genomic region in a cartilaginous fish species, we searched the elephant shark genome for *Dlx *- and URE2-like sequences. We found independent reads including putative Cm*Dlx1*, Cm*Dlx2*, Cm*Metapl1*, CmURE2 as well as CmI12a, a second *Dlx1/Dlx2 *regulatory element. After screening a BAC library for the putative Cm*Dlx1 *sequence, we isolated a BAC clone from which we could also PCR-amplify Cm*Dlx2*, the two putative enhancer sequences, CmURE2 and CmI12a, and exons 9 and 10 of the Cm*Metapl1 *gene (Figure 1).

**Figure 2:**
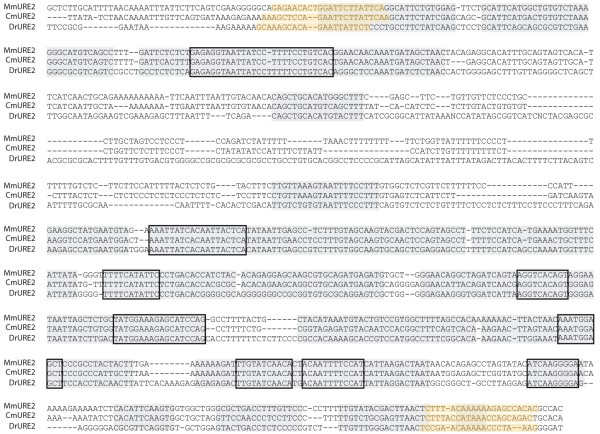
**Sequence alignment of the elephant shark (Cm), mouse (Mm) and zebrafish (Dr) URE2 sequences**. Alignable regions are highlighted grey, identical sequences (> 10 bp) between all three species are boxed, and the primers used to amplify these sequences are highlighted orange.

We produced an alignment of the elephant shark, mouse, and zebrafish URE2 sequences (1017 bp, Figure 2), approximately half of which could be aligned with no ambiguity (517 bp). The CmURE2 sequence closely resembles its mouse and zebrafish counterparts (Figure 2) with 85% identity between MmURE2 and CmURE2, 75% identity between MmURE2 and DrURE2, and 73% identity between DrURE2 and CmURE2. We then aligned the orthologous URE2 sequences extracted from the Ensembl Genome Browser (release 56, [31]) for three other tetrapod species (a frog, *Xenopus tropicalis *; a lizard, *Anolis carolinensis *; a bird, *Gallus gallus *) and three other teleost species (the medaka, *Oryzias latipes *; and two pufferfish, *Takifugu rubripes *and *Tetraodon nigroviridis *) (Additional File 1). Again, the elephant shark URE2 sequence was significantly (t-test; p < 0.05) more similar to tetrapod sequences (mean: 82.5%) than to teleost sequences (mean: 71.5%). When testing for relative substitution rates with the elephant shark as an outgroup, the null hypothesis of equivalent substitution rates could be confidently rejected (p = 0) when comparing zebrafish and mouse URE2 sequences or medaka and *Xenopus *URE2. In both cases a higher substitution rates was obtained in teleosts compared to tetrapods. These results strongly suggest that the elephant shark sequence is more similar to tetrapod sequences than to teleost sequences, most probably due to higher mutation rates in the latter clade.

### The elephant shark URE2 sequence acts as a forebrain regulatory element in transgenic zebrafish and mice

To determine if the CmURE2 sequence can act as a regulatory element and to compare its activity with its zebrafish and mouse counterparts, we prepared a series of reporter constructs in which the URE2 sequences are placed upstream of a cassette containing a β-*globin *minimal promoter and either the *GFP *or *lacZ *reporter gene. The resulting constructs were tested in both transgenic (Tg) zebrafish and mice.

**Figure 3:**
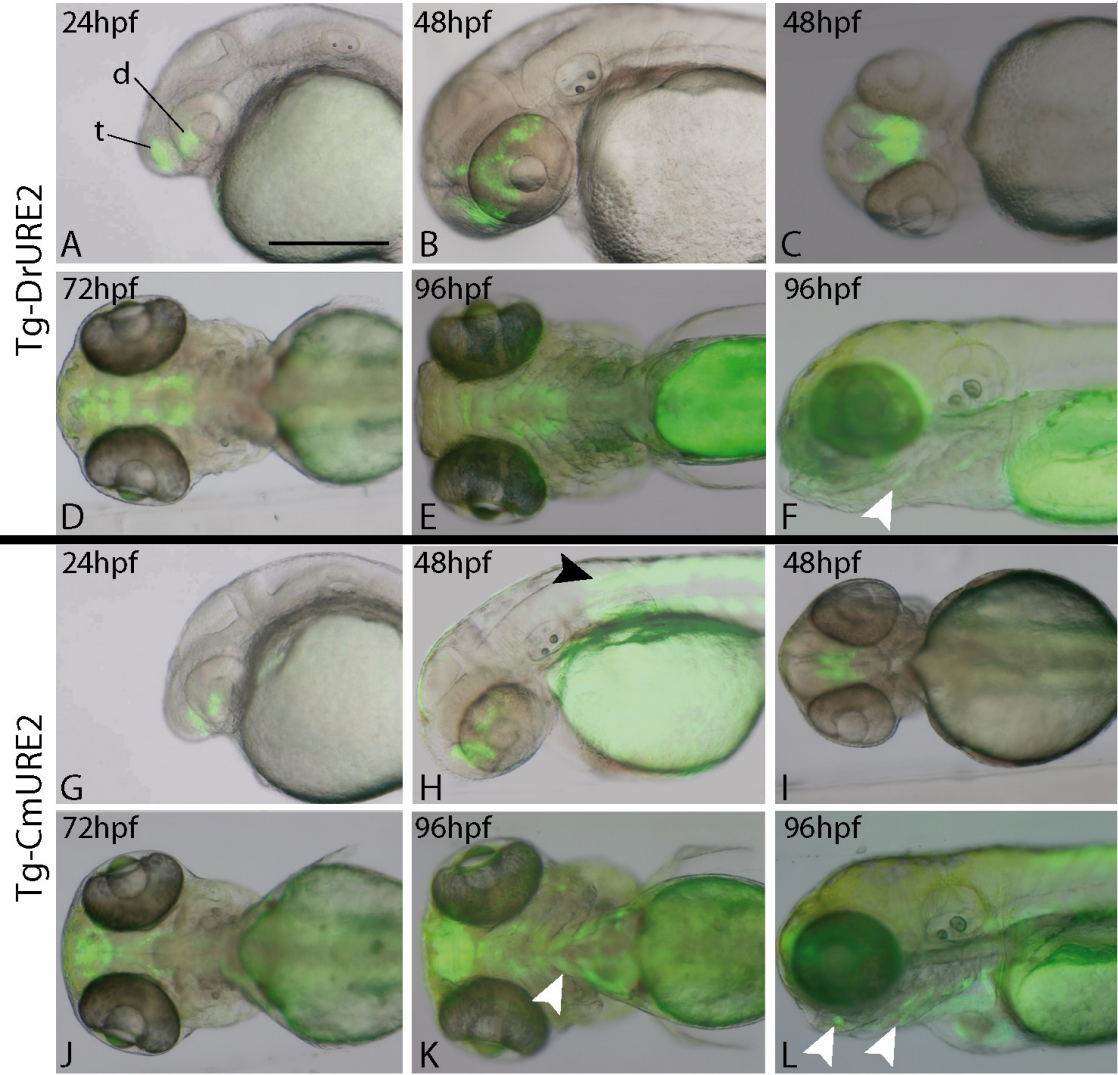
**Expression of URE2-GFP reporter transgenes in zebrafish**. The Tg-DrURE2 drives GFP expression in the forebrain starting at 24 hpf until at least 96 hpf (A-F). At 96 hpf GFP expression is also observed in the pharyngeal arches (E, F). The Tg-CmURE2 drives GFP expression in the forebrain at 24 hpf until at least 96 hpf (G-L). GFP expression is also first noticed in the somites and pharyngeal arches at 48 hpf (H) and 96 hpf (K, L), respectively. Panels A, B, F, G, H, L are lateral views, anterior to the left and dorsal to the top; panels C, D, E, I, J and K are ventral views. t: telencephalon; d: diencephalon. White arrowheads indicate pharyngeal arch expression and the black arrowhead indicates somite expression. Scale bar: 250 μm.

In zebrafish, the Tg-DrURE2 drove GFP expression in the telencephalon and diencephalon starting at approximately 24 hpf (Figure 3A). This expression was observed in two independent lines of transgenic zebrafish and persisted until 96 hpf (Figure 3B-F), a time where GFP expression was also noted weakly in the pharyngeal arches (Figure 3E, F). Similarly, the CmURE2 sequence targeted GFP expression to the forebrain of zebrafish embryos and larvae from 24 hpf until at least 96 hpf (Figure 3G-3K), with pharyngeal arch expression of the reporter transgene observed at the later time points in two independent transgenic lines (Figure 3K, L). Overall, the examination of live embryos indicated that the spatial distribution of the GFP protein was generally similar for both constructs suggesting similar activities for the elephant shark and zebrafish sequences in the brain and visceral arches (Table 1). One site of Tg-CmURE2 activity that is not observed with Tg-DrURE2, and is not consistent with endogenous *dlx1a *or *dlx2a *expression, is the somites at 2 dpf (Figure 3H, black arrowhead) continuing until at least 4 dpf (Figure 3L).

**Table 1:** Summary of URE2 reporter gene expression patterns

	Tg-DrURE2						Tg-CmURE2						Tg-MmURE2	
-	1 dpf	2 dpf	3 dpf	4 dpf	E11.5		1 dpf	2 dpf	3 dpf	4 dpf	E11.5		2-4 dpf	E11.5
	
Telencephalon	+	+	+	+	+		+	+	+	+	+		+	+
Diencephalon	+	+	+	+	+		+	+	+	+	+		+	+

Visceral arches	-	-	+	+	-		-	-	+	+	-		+	+

Dorsal root ganglia	na	na	na	na	-		na	na	na	na	+		na	+

Somite muscles	-	-	-	-	na		-	+	+	+	na		na	na

Fin/limb buds	-	-	-	-	-		-	-	-	-	-		na	+

Summary of reporter gene expression patterns under regulation by the zebrafish, elephant shark or mouse URE2 sequences (respectively Tg-DrURE2, Tg-CmURE2, Tg-MmURE2) in zebrafish embryos staged from 1 to 4 days post-fertilization (dpf) in case of stable transgenic lines, 2 to 4 dpf in case of primary transgenic expression (Tg-MmURE2) and in mouse embryos aged E11.5 (one stable transgenic line for Tg-MmURE2, primary transgenic expression for Tg-DrURE2 and Tg-CmURE2).

**Figure 4:**
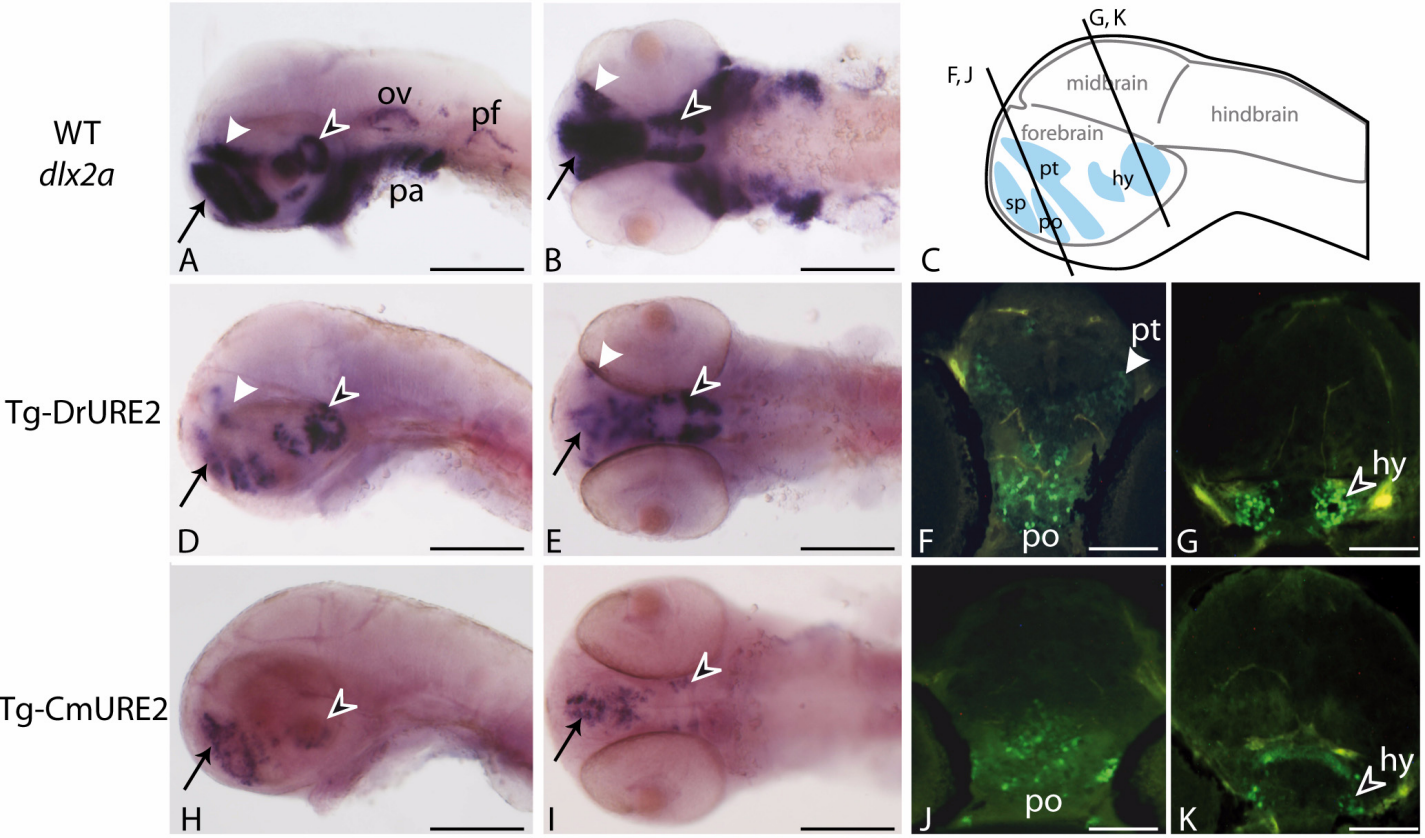
**Expression of URE2-GFP reporter constructs in the brain of 48 hpf zebrafish**. Expression patterns obtained by in situ hybridization using a *dlx2a *cDNA probe in wild-type embryos (A, B) or a *GFP *probe in Tg-DrURE2 (D, E) and in Tg-CmURE2 embryos (H, I). Immunolocalization of GFP proteins on sectioned embryos of the Tg-DrURE2 (F, G) and Tg-CmURE2 (J, K). Expression in the telencephalon is comparable for the endogenous gene and the two transgenes (black arrow in A, B, D, E, H, I). Expression in the dorsal domain of the prethalamus (white arrowhead) in Tg-DrURE2 for *gfp *mRNA (D, E) and GFP proteins (F) is not observed in Tg-CmURE2 (H-J). Expression of GFP in the hypothalamus (black arrowhead) was restricted to lateral cells in Tg-CmURE2 (H, I, K) compared to Tg-DrURE2 (D, E, G). Panels A, D, H are lateral views, B, E, I are ventral views. Plan for the transversal sections presented in F-G and J-K are localized on the scheme in panel C. Blue domains in the scheme are the forebrain expression domains described for *dlx *genes: the telencephalic domain being the subpallium (sp, black arrow); the diencephalic domains being the preoptic area (po), prethalamus (pt, white arrowhead) and hypothalamus (hy, black arrowhead). Scale bars: A, B, D, E, H, I, 250 μm; F, G, J, K, 50 μm.

We then examined the activity of the URE2 elements in the forebrain in greater detail and compared this with the endogenous expression of the zebrafish *dlx1a/2a *genes. The endogenous *dlx *expression domains correspond to the subpallium of the telencephalon and to specific regions of the diencephalon (preoptic area, prethalamus, and hypothalamus) (Figure 4A-C) [30]. Comparative *in situ *hybridization analysis of the *GFP *transcripts identifies highly comparable expression with endogenous *dlx2a *(Figure 4A, B) and *GFP *in Tg-DrURE2 embryos (Figure 4D, E). However differences in transgene expression could be pointed in the Tg-CmURE2 line with no apparent detection of *GFP *expression in the prethalamus, as well as very restricted expression in the hypothalamus (Figure 4H, I). Anti-GFP immunohistochemistry on sections of transgenic embryos confirmed that the prethalamus expression was completely absent from Tg-CmURE2 embryos, while expression in the pre-optic area is comparable between Tg-CmURE2 and Tg-DrURE2 (Figure 4F, J). More posterior in the hypothalamus, the transgene was expressed only in a restricted lateral domain in the Tg-CmURE2 transgenic line, while the GFP expression domain was larger in the Tg-DrURE2 line (Figure 4G, K). Immuno-localization of the GFP in these two lines also allowed us to detect GFP in the muscles associated to the visceral arches, as well as muscles in the tail (data not shown), consistent with the fluorescence patterns described in Figure 3. Examination of primary transgenic zebrafish obtained with a similar construct containing the mouse MmURE2 sequence indicates that it behaves similarly to its elephant shark and zebrafish counterparts with expression in the telencephalic and diencephalic domains (Additional File 2). Notably, the transgene could also be detected in visceral arches at 5 dpf, in a pattern similar to that observed for Tg-DrURE2 and Tg-CmURE2 (Additional File 2C).

**Figure 5:**
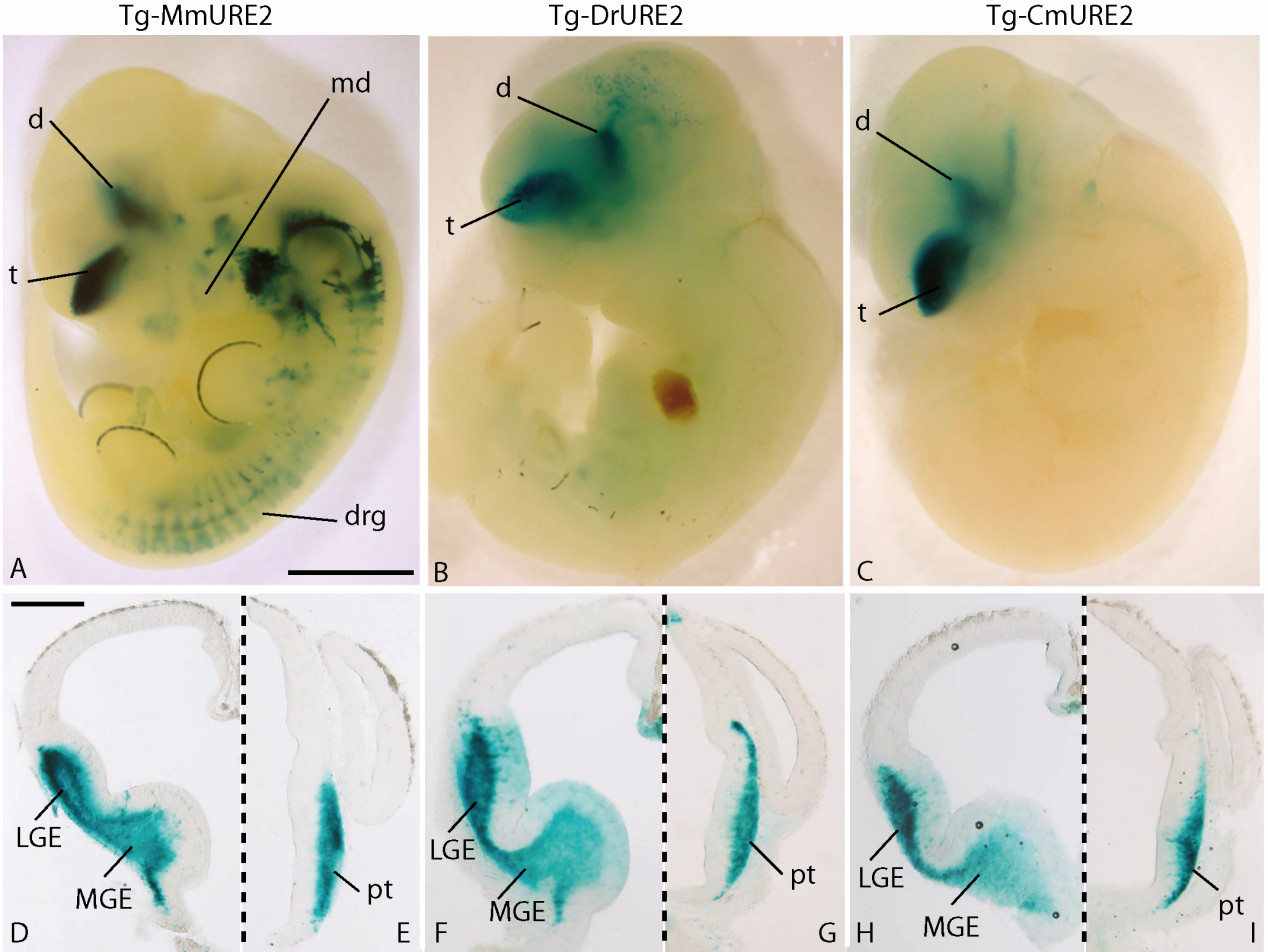
**Expression of URE2-lacZ reporter constructs in E11.5 mouse embryos**. Lateral views of a E11.5 embryo from a Tg-MmURE2 transgenic line (A) and primary transgenic embryos for Tg-DrURE2 (B), or Tg-CmURE2 (C). Dissected brains were sectioned at the level of the telencephalon (D, F, H) and diencephalon (E, G, I) and detailed expression of the transgene was found comparable for Tg-MmURE2 (D, E), Tg-DrURE2 (F, G), and Tg-CmURE2 (H, I). Panels E-J are halves of transversal sections, dorsal is up, left side is shown in E, G, I, right side in F, H, J, sagittal plan figured in dashed line. d: diencephalon; drg: dorsal root ganglia; pt: prethalamus; t: telencephalon. Scale bars: 1 mm in A-C, 500 μm in D-I.

Similar constructs using *LacZ *as the reporter gene were tested in primary transgenic mouse embryos at E11.5. The three URE2 enhancers had very similar activities in the forebrain (Figure 5; Table 1). All three URE2 sequences targeted expression to the telencephalon and diencephalon (Figure 5A-I). Forebrain expression of the reporter constructs was observed in 4/5 and 4/4 primary transgenic embryos obtained with CmURE2-*lacZ *and DrURE2-*lacZ *, respectively (Additional File 3). Outside the forebrain, the mouse and elephant shark URE2 sequences showed more similarities in their activities compared to zebrafish URE2: both CmURE2 and MmURE2 could target expression to the dorsal root ganglia in primary transgenic embryos (CmURE2-lacZ, n = 1/5; Additional File 3) or in two independent transgenic lines (MmURE2-lacZ, Figure 5A and 19). The DrURE2-lacZ transgene was not expressed in the dorsal root ganglia (n = 0/4). The mouse URE2 element was the only one able to target expression to the branchial arches and to the apical ectodermal ridge of limb buds.

## Discussion

### Locus and sequence conservation among jawed vertebrates

In this study we identify a conserved regulatory region associated with *Dlx *genes in the elephant shark. Conserved synteny could not be precisely determined because the elephant shark genome is not assembled. However, the sequence identified as CmURE2 is located on the BAC clone containing the elephant shark *Dlx1 *, *Dlx2 *and *Metapl1 *genes, similar to what is observed in all other jawed vertebrates for which genomic data are available (Ensembl Release 56, [31]). The putative conservation of the bigene cluster organisation between bony vertebrates and cartilaginous fish is consistent with the hypothesis that an ancestral chordate bigene cluster has been duplicated twice before the radiation of jawed vertebrates [32,33]. The identification of a URE2 sequence in vicinity of the elephant shark *Dlx1 *and *Dlx2 *genes also indicates this locus was linked to these genes in the ancestor of all jawed vertebrates. A search for sequences orthologous to this URE2 enhancer in species outside of jawed vertebrates did not yield any significant hit with the BLASTn tool from the NCBI sequence browser [34] or the BLAT tool in the UCSC genome browser [35] on the lamprey (*Petromyzon marinus *), tunicate (*Ciona intestinalis *) or lancelet (*Branchiostoma floridae *) genomes, even though *Dlx *genes have been identified in these organisms and bigene *Dlx *tandems are present in lamprey and tunicate [15,33,36] . The high level of URE2 sequence conservation observed in jawed vertebrates suggests that it has evolved under high evolutionary constraints and that its enhancer activity likely emerged in the jawed vertebrate ancestor, after divergence of the cyclostomes, i.e., more than 400 Myrs ago. Interestingly, this reflects the trend observed for a number of other vertebrate CNEs [5].

### Enhancer activity, conservation, and variation

The most prominent expression domain of a transgene under the regulation of MmURE2 or DrURE2 has been shown to be the forebrain, more precisely in the ventral telencephalon and diencephalon, in agreement with the pattern of endogenous expression of *Dlx1 *and *Dlx2 *genes [17]. Both zebrafish transgenic lines obtained with DrURE2 and CmURE2 driving expression of GFP produced comparable expression patterns, suggesting the regulatory activity of these orthologous sequences in developing forebrain and visceral arches is likely to be conserved. This overall conservation of the enhancer activity, along with a high level of identity between the two sequences, implicates conservation of transcription binding sites allowing the CmURE2 to retain activity in the developing zebrafish and mouse brain. The results obtained with the DrURE2 transgenic zebrafish lines show that the activity of this enhancer recapitulates part of the *dlx1a/dlx2a *endogenous expression pattern in the forebrain. Similarly MmURE2 transgenic mouse lines recapitulate endogenous *Dlx1/Dlx2 *expression in this domain [18]. Thus, the conservation of URE2 regulatory sequences correlates with conservation of their activity.

However, differences could be identified in the diencephalic expression domains (prethalamus and hypothalamus) between the two transgenic lines. These discrepancies suggest that while the overall activity is conserved, the URE2 enhancer also shows some degree of modularity across the vertebrate phylogeny. Accordingly, the differences between CmURE2 and DrURE2 sequences could account for the differential expression pattern in the prethalamus and hypothalamus between different vertebrate species, whereas these sequence differences do not modify the enhancer activity in the telencephalon. In turn, these results suggest that these two *Dlx *gene expression domains (telencephalon and diencephalon), even though both regulated by a unique functional URE2 enhancer, are perhaps the result of two distinct genetic pathways.

The DrURE2 and CmURE2 sequences also drive expression in the developing telencephalon and diencephalon of transgenic mice, a pattern comparable to the endogenous MmURE2 enhancer. Again, this highlights the conservation of the regulatory cascade leading to *Dlx *gene expression in the developing forebrain of mice. In this species, the function of URE2 in the forebrain seems to be completely conserved despite variation in the sequences, which highly contrasts with our results from transgenic assays in zebrafish where expression in the diencephalon seems to be sensitive to sequence variations. Our results suggest that a distinct genetic pathway is specifically involved in teleost diencephalon development that would not be shared with mouse. This new pathway could have emerged after the additional genome duplication event occurring before teleosts radiation, which seeded many paralogous developmental genes that have the potential to be co-opted (or recruited) as new independent upstream signals interacting with the zebrafish URE2 enhancer. However, we cannot rule out the possibility that subtle changes in zebrafish transcription factor binding specificity may account for the apparent divergence of the CmURE2 enhancer function in the zebrafish forebrain, compared to the mouse forebrain.

The URE2 sequences studied here are also able to drive expression in the developing branchial arches. More specifically, the MmURE2 can drive expression in the hyoid arch mesenchyme in transgenic mice, while DrURE2 and CmURE2 did not produce any branchial signal in our primary transgenic embryos. DrURE2 and CmURE2, and possibly MmURE2, were able to drive GFP expression in muscles of the growing mandible and of the posterior-most visceral arches in transgenic zebrafish. The GFP fluorescence pattern obtained in zebrafish visceral arches (mandible and branchial arches shows expression in the muscles associated with the arches, rather than expression in the chondrogenic mesenchyme, where the *dlx *genes are known to be transcribed [37]. This expression pattern is unlikely to be an insertion artefact because it could be observed in two independent insertions for both DrURE2 and CmURE2 transgenic lines. GFP expression in arches muscles could be the result of endogenous URE2 activity that was not reported in previous studies on *dlx *gene expression patterns [37]. Alternatively, detection of GFP in these muscle cells could be the result of GFP stability in cell lineages derived from cells where *dlx *genes are endogenously transcribed.

Lineage-specific modifications may account for the differences observed in the branchial arches between mouse and zebrafish, such as the mammal-specific loss or teleost-specific gain of upstream signal targeting in the visceral arch mesenchyme. These hypotheses could be tested by biochemical and molecular techniques comparing the ability of different activators from the different lineages to enhance expression in response to these signals on the orthologous CREs. In this respect, it will be interesting to characterize the various transcription factors that interact with the MmURE2 sequence and contribute to expression in the brain or visceral arches. In mouse assays, the Tg-MmURE2 and Tg-CmURE2 sequences were able to consistently drive expression not only in the developing brain, but also in the dorsal root ganglia. None of the Tg-DrURE2 primary embryos (n = 0/4) had expression in these structures, suggesting that MmURE2 and CmURE2 share some enhancer activity that has been lost by DrURE2 consistent with absence of *dlx1a/dlx2a *expression in dorsal root ganglia.

### URE2 evolution in vertebrates

As no expression data is available from the elephant shark, we cannot correlate the CmURE2 enhancer activity to the endogenous *Dlx1 *and *Dlx2 *expression patterns. It is therefore difficult to propose an overview of the evolution of the URE2 enhancer in vertebrates. However, our results show that the genomic organisation of the *Dlx1/Dlx2 *bigene cluster with a URE2 sequence in the vicinity is conserved amongst all jawed vertebrates. The three URE2 sequences coming either from a cartilaginous fish (the elephant shark), a teleost fish (the zebrafish), or a tetrapod (the mouse) are able to drive expression in the forebrain with apparent complete robustness. These results highlight the strong selective constraint that may have acted against the modification of the regulatory sequences and the *trans *-activating protein domains, which interact with these enhancers, during jawed vertebrate evolution. However our results also show that URE2 enhancer activity in visceral arches and diencephalon is only partially conserved and has accumulated evolutionary modifications leading to variations from one organism to another. In particular, the lack of regulatory activity of CmURE2 and DrURE2 in the visceral arches of the mouse could be the result of lineage-specific sequence modification in transcription factor binding sites during tetrapod evolution, possibly leading to modifications of the regulatory cascade involving the URE2-*Dlx1 *-*Dlx2 *module in the branchial arches.

## Conclusions

As a chondrychtyan, the elephant shark provides a useful model to carry out comparative studies with jawed vertebrates to evaluate the relative contributions of changes in coding sequences and in CREs, These changes may have lead to morphological innovations, such as the tripartite brain and branchial arches of jawed vertebrates. The use of the elephant shark had been limited to comparative DNA sequence analysis [21]. Here, we have shown that CREs from the elephant shark can be successfully tested in teleost and tetrapod experimental models. Whereas transgenes with elephant shark CREs cannot yet be tested endogenously, as transgenesis in this species has yet to be developed, it may be possible to obtain gene expression data in elephant shark for comparative purposes. Such expression studies would further increase the usefulness of the elephant shark in evolutionary developmental biology as an outgroup of bony vertebrates (zebrafish and mouse) showing not only a conserved genome structure but also, as highlighted here, conserved gene regulatory mechanisms.

## Methods

### Sequence identification and manipulation

The sequence of the previously identified regulatory sequence URE2 from the mouse was blasted against the 1.4 × coverage survey-sequencing data of the elephant shark genome [38]. One significant hit allowed us to identify the homologous sequence to the mouse URE2 (MmURE2) in the elephant shark genome (CmURE2). A BAC from the elephant shark genome library (IMCB_Eshark BAC library) was isolated by 3-step PCR screening of the pooled BAC DNA using primers for the elephant shark *Dlx1 *gene (CTCCTCTCCCTTTCAGCAGCAG and ATTACCTGTGTCTGTGTGAGTCC). This BAC was used as a template for PCR with primers designed for *Dlx2 *gene (GAGAAATGCCGACAGATCAGCTC and CCACCATAGGCTGATGTTGTATG) and the CmURE2 enhancer (AAAGCTCCAGAATTCTTATTCA and GTCTGCTGGTTTATGGTAAAG) and the *Metapl *gene (exons 9/10: GCTCGAACTGGGCTGATCTA and TGGACAGCAATTTCCAATGA: exon 7: AATGGACTGCAAGTTTGCCC and GCAGCCCTTATCCAGTAGAA) that were hypothesized to be in a region of conserved synteny with the URE2 sequence in other vertebrate genomes (Figure 1).

Orthologous URE2 sequences were retrieved from the Ensembl genome browser (release 56) by blast with the zebrafish URE2 sequence (DrURE2) against the genome of seven other species: mouse, *Mus musculus *; chicken, *Gallus gallus *; anole lizard, *Anolis carolinensis *; xenopus, *Xenopus tropicalis *; medaka, *Oryzias latipes *; fugu *Takifugu rubripes *; Tetraodon *Tetraodon nigroviridis*. The sequences were first aligned with ClustalW implemented in BioEdit and the alignment was then refined by eye (total 1097 nucleotidic sites, see Suppl. Figure 1) [39]. In a zebrafish/mouse/elephant shark sequences comparison, we defined by eye the unambiguously aligned regions within the alignment of the three sequences (see Figure 2, final 578 bp). Percentages of identity were calculated and conserved regions were identified by BioEdit software (minimum segment length, 10 bp; gaps limited to 2 by segment and only 2 consecutive gaps allowed). In the comparisons of all nine species, only positions with gaps were excluded (final 705 bp) before the percentages of identity were calculated between two sequences or before relative substitution rate tests were evaluated with the MEGA software [40].

### Transgene constructs

For transgenic zebrafish, the URE2 element was inserted into the multiple cloning site of a vector that contained a β-globin minimal promoter-GFP cassette. The URE2 sequence is located immediately upstream of the β-globin-GFP fragment and the resulting URE2-β-globin-GFP DNA fragment is flanked at both ends by Tol2 recombinase recognition sequences [41]. For transgenic mice, the URE2 element was inserted into the multiple cloning site of the p1230 construct [42] Microinjection of transgene constructs into fertilized mouse eggs and production of transgenic mice were carried out as previously described [16]. For the production of transgenic zebrafish, approximately 125 ng of a tol2 *transposase *mRNA synthesized *in vitro *with 50 ng/ml of DNA construct was co-injected along with the transgene constructs into fertilized zebrafish embryos at the one-cell stage. At least two independent lines of transgenic zebrafish were produced, unless otherwise indicated.

### Animals

Zebrafish were raised at 28°C under a 14:10 hour light-dark cycle as previously described [43]. All animal manipulations were preformed according to guidelines from the Canadian Council for Animal Care.

### In situ hybridization

Zebrafish for RNA *in situ *hybridizations and fluorescence imaging were treated with 0.0015% 1-phenyl 2-thiourea (PTU) to inhibit melanogenesis. Whole mount *in situ *hybridizations were carried out following previously described protocol with digoxygenin-labelled cRNA probes synthesized on previously described templates *dlx2a *[29] and *gfp *[44]. Coloration was achieved with Nitro-Blue Tetrazolium Chloride (NBT) and 5-Bromo-4-Chloro-3'Indolyphosphate P-Toluidine Salt (BCIP) solution [45].

### Microscopy and Immunohistochemistry

Whole mount images were taken on a Nikon NBZ 1500 dissecting microscope with a Nikon DXM 1200 C digital camera. Immunohistochemistry was carried out as previously described [17], on cryosections at a thickness of 10 μm. Primary antibody: Rabbit anti-GFP (1:1000, Invitrogen, A-11122). Secondary antibodies: Goat anti-rabbit Alexa Fluor488 (1:300, Invitrogen, A11008). Signals were visualized on a Nikon Eclipse E3600 stereomicroscope for fluorescent stains.

## Authors' contributions

RBM contributed to the production of constructs, transgenic mice and zebrafish lines/primary embryos, to the analysis of transgenic lines and to the writing of the manuscript; MDT contributed to sequence analyses, transgenic animal analyses and to the writing of the manuscript; KM contributed to the production of gene constructs and transgenic zebrafish lines; LP contributed to the production of constructs used in transgenesis and of the mouse transgenic line; BHT screened and identified the elephant BAC clone; BV provided access to elephant shark sequences and to BAC clones, contributed to the analyses of the data and to the writing of the manuscript; ME contributed to the design of the study, to the analyses of the data and to the writing of the manuscript. All authors have read and approved the final manuscript.

## Supplementary Material

Additional file 1 **Jawed vertebrate URE2 sequences alignment and similarity**. A. Alignment of URE2 sequences from tetrapods (Mm: *Mus musculus *; Gg: *Gallus gallus *; Ac: *Anolis carolensis *; Xt: *Xenopus tropicalis *) and teleosts (Dr: *Danio rerio *; Ol: *Oryzias latipes *; Tn: *Tetraodon nigroviridis *; Tr: *Takifugu rubripes *) with elephant shark (Cm) URE2 sequence highlighted grey.
B. Percentage of identity between two sequences after gap exclusion in the previous alignment Click here for file

Additional file 2 **Primary transgenic zebrafish embryos with *gfp *expressed under MmURE2 sequence**. GFP fluorescence could be detected in the forebrain (fb) of primary transgenic zebrafish at 2 dpf (A) and 3 dpf (B) after injection of the construct. GFP fluorescence was also detected in the visceral arches (va) of 4 dpf old embryos after injection (C). Click here for file

Additional file 3 **Primary transgenic mice with *LacZ *expressed under CmURE2 (A-E), DrURE2 (F-H) sequences to be compared with *LacZ *expression in a stable transgenic line under MmURE2 regulation (G), at E11.5**. d: diencephalon, drg: dorsal root ganglia, md: mandibular arch, t: telencephalon. Click here for file
