# Supplementary material for: Functional conservation of a forebrain enhancer from the elephant shark (Callorhinchus milii ) in zebrafish and mice
Source: BMC Evol Biol. 2010 May 26;10:157. doi: 10.1186/1471-2148-10-157 (PMC2891724; doi:10.1186/1471-2148-10-157)
Supplement: Additional file 1 — Jawed vertebrate URE2 sequences alignment and similarity. A. Alignment of URE2 sequences from tetrapods (Mm: Mus musculus ; Gg: Gallus gallus ; Ac: Anolis carolensis ; Xt: Xenopus tropicalis ) and teleosts (Dr: Danio rerio ; Ol: Oryzias latipes ; Tn: Tetraodon nigroviridis ; Tr: Takifugu rubripes ) with elephant shark (Cm) URE2 sequence highlighted grey. B. Percentage of identity between two sequences after gap exclusion in the previous alignment [file 1471-2148-10-157-S1.PDF]

A

```
               ....|....| ....|....| ....|....| ....|....| ....|....| ....|....| ....|....| ....|....| ....|....| ....|....|
                   5         15         25         35         45         55         65         75         85         95
MmURE2         GCAGAGAACA CTGGATTCTT ATTCAGGCAT TCTGTGGA-G -TTCTGCATT CATGGCTGTG TCTAAAGGGC ATGTCAGCCT TT--GATTCT CTCTGAGAGG
GgURE2         GCACAGAACA CTAGATTCTT ATTCAAGCAT TCTATCGA-G C-TCTGCATT CATGGCTGTG TCTAAAGGGC ATGTCAGCCT TT--GATTCT CTCTGAGAGG
AcURE2         GCACCGAAGA CGAGATTCTT ATTCAAGCAT TCTCCTGA-G CA-CTGCATT CATGGCTGTG TCTAAAGGGC ATGTCAGCCT TT--GATTCT CTCTGAGAGG
XtURE2         GCAGAGTGGA GCAGATTCTT ATTTAGGCAT TCTATGGA-G C-TCTGCATT CATAGCTGTG TCTAAAGGGC ATGTCAGCCT TT--GATTCC TTCTGAGAGG
CmURE2         AAAAGCTCCA --GAATTCTT ATTCAAGCAT TCTATCGA-G CAGC-GCATT CATGACTGTG TCTAAAGGGC ATGTCAGTCT TTT-GATTCA CTTTGAGAGG
DrURE2         GCAAAGCACA --GAATTATT CTCCCTGCCT TCTATCAA-G CTCCTGCATT CAGCAGCGCG TCTGAAGGGC GCGTCAGTCC GCCTGCCTCT CTCAGAGAGG
OlURE2         GCAAAGAACA --AAATTATT CCTCAAGCTT TCTATCAAAG CCTCTGCATT CATGGCAAAG TCTAAAGGGC ATGTCAGTCC TCCTGCC--T CTTAGAGAGG
TnURE2         GCAAGGAAGA --AAATTATT CCTCCAGCTT CCTATTGA-G CTTCTGTATT CACGGCCAAG TCTAAAGGCC ATGTCAGTCC TCCTGCC--T CTCAGAGAGG
TrURE2         GCAAAGAACA --AAATTATT CCTCAAGCTT TCTATCAA-G CTTCTGCATT CACGGCCAAG TCTAAAGGGC ATGTCAGTCC TCCTGCC--T CTGAGAGAGG

               ....|....| ....|....| ....|....| ....|....| ....|....| ....|....| ....|....| ....|....| ....|....| ....|....|
                   105        115        125        135        145        155        165        175        185        195
MmURE2         TAATTATCC- TTTTCCTGTC ACGGAACAAC AAATGATAGC TAACTACAGA GGCACATTTG CAGTAGTCAC A-TTCATCAA CTGCAGAAAA AAAAAA-TTC
GgURE2         TAATTATCC- TTTTCCTGTC ACGGAACAAC AAATGATAGC TAACTACAGA GGCACATTTG CAGTAGTCAC A-TTCATCAA CTGCAGAA-A AAAAAA-TTC
AcURE2         TAATTATCC- TTTTCCTGTC ACGGAACAAC AAATGATAGC TAACTACAGA GGCACATTTG CAGTAGTCAC A-TTCATCAA CTGCAGGGGA AAAAAA-TTC
XtURE2         TAATTATCC- TTTTCCTGTC ACGGAACAAC AAATGATAGC TAACTACACA AGCGCATTTG CGGTAGTCAC A-TTCATCAA CTGCAGGGAA AAAAAA-TTC
CmURE2         TAATTATCC- TTTTCCTGTC ACTGAACAAC AAATGATAGC TAACT---GA GGCACATTTG CAGTAGTTGC A-TTCATCAA TTGCTA---A AAAAAA-TTG
DrURE2         TAATTATCCT TTTTCCTGTC ACAGGGCTCC AAATGATCTC TAACCACTGG GGAGCTTTGT TAGGGGCTCA GCTTTGGCAA TAGGAAGTCG AAAGAGCTTT
OlURE2         TAATTATCC- TTTTCCTGTC AGGGAATAAC AAATGATAGT CAACTATGGG GGTGCAGTTT CAGGAGCTGA AATTTGTTGA TAGGAAGGAG AAGGAGGTTT
TnURE2         TAATTATSCC TTTTCCTGTC AGAGGACAAC AAATGATCGT CGACGAC-GG GGCGCATTTT CTGGAGCTGA AATTTGTCAG TTGCTAGAAA AAGAGGGTTT
TrURE2         TAATTATCC- TTTTCCTGTC AGAGACCAAC AAATGATCGC CAACTAT-GG GGC--ATTTT CTGGAGCTGA A-TTTGTCAG TTGGGAGAAA AAGGGGATTT

               ....|....| ....|....| ....|....| ....|....| ....|....| ....|....| ....|....| ....|....| ....|....| ....|....|
                   205        215        225        235        245        255        265        275        285        295
MmURE2         AATTTAATTG TACAACACAG CTGCACATGG GCTTTC---- ---GAGC--T TC---TGTTG TTCTCCCTGC ---------- ---------- ----------
GgURE2         AATTTAATTG TGCAACACAG CTGCACATAG GCTTTTT--- ---GAGCATT TC---TGTTG TTCTCCCTGT ---------- ---------- ----------
AcURE2         AATTTAATTG TACAACACAG CTGCACATAG GCTTTTT--- ---GAGCATT TC---TGTTG TTCTCTCTGT ---------- ---------- ----------
XtURE2         AGTTTAATTG TACAACCCAG CTGCACATGA GCCTTT---- ---CAGCATA TC---TGTTG TTCTCTCTTT ---------- ---------- ----------
CmURE2         AATTTAATTG TGTAACACAG CTGCATGTCA GCTTTTAT-- ---CA-CATC TC---TCTTG TACTGTGTGT ---------- ---------- ----------
DrURE2         AATTT---TC AGA----CAG CTGCACATGT ACTTTCATC- --GCGGCATT ATAAANCCAT ATAGCGGTCA TCNCTAC--- ---------- ----------
OlURE2         AATTT---TC TGG----CAG CTGCACATCT ACTCTTATCT GGGTTTTTTT TTTGGTCAGT TTTTTGCCTT GTGCAG---- ---------- ----------
TnURE2         AATT---TTC TGTTTC-CCA CTGGTCAT-T TCTT-CTTTT TTTTCCTTTT TCTCCACACT CTCAGTCTGC CGGAG----- ---------- -------CAG
TrURE2         AATT---TTC TGG----CAG CTGTACATCT ACTTTCATGT TTTTCCCCCC ACTGGTCATT TCTTGTTTTT TGGGGGGGGG GTTCCCCCAC TATCCTGCAC

               ....|....| ....|....| ....|....| ....|....| ....|....| ....|....| ....|....| ....|....| ....|....| ....|....|
                   305        315        325        335        345        355        365        375        385        395
MmURE2         ---------- --------CT TGCTAGTCCT CCCT------ CCAGATCTAT TTTT------ -TAAACTTTT TTTT---TTC TGGTTATTTT TTCCCCT---
GgURE2         ---------- --------CT TGCTATTCCT CCCT------ CCAGATCTAT TTTT------ -TAAACTTTT TTT------C TGGTTATTTT TTCCCCC---
AcURE2         ---------- --------CT TGCTATTCCT CCCTA----- CCAGATCTAT TTTT------ -TAAACTTTT TTTTCCTTCC TGCTTATTTT TCCCTCTCCC
XtURE2         ---------- --------CT TCTTA-CACT CCTT------ CCTGGTCTTT TTTTTTTTCC CTAAACTTTT ---CCCC-CT TGATTATTTT TTTTCCC---
CmURE2         ---------- --------CT GGTTCTCTTT CCCT------ CTATATATCC ATTTTCTTAT T--------- --------CC ATCTTGTTTT TTTTTCT---
DrURE2         -GAGCGCACG CGCGCACTTT TGTTTGTGAC GTGGGGCCGC GCGCGCGCGC CTGCCTGTGC ACGGCCTCCC CGCATTAGCA TATTTATTTA TAGACTTACA
OlURE2         --AGCAGTGC TG-------- -TTTGTTTAC GTGTGCGAGC TTGTGAGGGT GTATTTATGT G------CCC TGCATTAACA TGCCTATTTA TAATCCTTCA
TnURE2         AGAGCGCTCC TGGGCCATTT TTTTGTG--C CTGTGAA-GT CTCTGAGGGT GTATTTATGC GA-----TCC TACATTAACA TACCTATTTA TAGTCCTACA
TrURE2         AGAGAGCT-C TGCCGGGGCT GTTTGTGCGC CTGTGAA-GC CTCTGAGAGT GTATTTATGT G------CCC TACATTAACA TACCTATTTA TAGTCCTACA
```

```
                ....|....| ....|....| ....|....| ....|....| ....|....| ....|....| ....|....| ....|....| ....|....| ....|....|
                     405        415        425        435        445        455        465        475        485        495
MmURE2          ---------- ---TTTTTTG TCTC-----T TCTTCCATTT TTACTCTCTG ----TACTTT CTTGTTAAAG TAATTTTCCT TTGTGGCTCT CGTTCTTTTT
GgURE2          ---------- ----TTTTTG TCTC-----T TCTTCCATTT TTACTCTCTG ----TACTTT CTTGTTAAAG TAATTTTCCT TTGTGGCTCT CATTCTTTTT
AcURE2          CCTCCCCCCT CTTTTTTTTG TCTC-----T TCTTCCATTT TTACTCTCTG ----TACTTT CTTGTTAAAG TAATTTTCCT TTGTGGCTCT CATTCTTCTC
XtURE2          ---------- ----ATTTTG TCTA-----T TCCTCCATTT TTTTTTTTTA CTC-TACTTT CTTGTTAAAG TAATTTTCCT TTGTGGCTCC TTTTTTTTTC
CmURE2          ---------- -----CTTTA CTCT------ CTCTCCCTCT CTCCCTCTCT C---TCCTTT CCTGTTAAAG TAATTTTCCT TTGTGAGTCT CATTCTT---
DrURE2          CTTTTCTTAC AGTCATTTTT GCGCAA---- ---------- --CAATTTT- CACACTCGAC ATTGTCTGTG TAATTTCCCT TTCAGTGTCT CTCTGTTTTC
OlURE2          CTTT-CTTAC AGTCATCTTT TTT------T TTCTGTTGT- GACCATTTCA CAGATTATAT ATTGTCCAAG TAATTTCCCT TTCAGCATCT GTGTTTTTTA
TnURE2          CTTT-CTTAC ATTCATCTTT TTTTTTTTTT TTTCTGTTGT GACCATTTCA CAGATTATAT ATTGTCCGAG TAATTTCCCT TTCAGCATCT GTGTTTTTTA
TrURE2          CTTT-CTTAC AGTCATCTTT TT-------- TTTCTGTTGT GACCATTTCA CAGATTATAT ATTGTCCAAG TAATTTCCCT TTCAGCATCT GTGTTTTTTA

                ....|....| ....|....| ....|....| ....|....| ....|....| ....|....| ....|....| ....|....| ....|....| ....|....|
                     505        515        525        535        545        555        565        575        585        595
MmURE2          TCC------- -----CCATT ----GAAGGC TATGAATGTA G--AAAATTA TCACAATTAC TCATATAATT GAGCC-TCTT TGTAGCAAGT ACGACTCCAG
GgURE2          TCC------- -----CCATT ----GAAGGC TATGAATGTA G--AAAATTA TCACAATTAC TCATATAATT GAGCC-TCTT TGTAGCAAGT GCAACTCCAG
AcURE2          CCCCCC---- -CCCCCCATT ----GAAGAC TATGAATGTA G--AAAATTA TCACAATTAC TCATATAATT GAGCC-TCTT TGTAGCAAGT GCAACTCCAG
XtURE2          TCTCTTGCTT CTCCCCCATT ----GAAGGA TATGAATGTA G--AAAATTA TCACAATTAC TCATATAATT GGGCC-TCTT TGTAGCAAGT GCAACTCCTG
CmURE2          ---------- -----GATCA AGTAAAGGTC CATGAATGGA CT--AAATTA TCACAATTAC TCATATAATT GAACCTCCTT TGTAGCAAGT GCAACTCCAG
DrURE2          TCCTCTCTTT CCTTCCCTTT CAGAAAGAGC CATGAATGGA TGGAAAATTA TCACAATTAC TCACATAATT GAGCCGTCTT TGTGGCAAGT GCAGCTCGAG
OlURE2          C--------- ------CTTT CAGAGAAAGC CGTGAATGGA GGGGAAATTA TCACAATTAC TCATATAATT GAGCCGTCTT TGTAGCAAGT GCGGCTTCAG
TnURE2          C--------- ------CTTT CAGAGAAAGG AGTGAATGGA GGG-AAATTA TCACAATTAC TCATATAATT GAGCCGTCTT TGTAGCAAG- GCAGCTTCAG
TrURE2          C--------- ------CTTT CAGCGAAAGG AGTGAATGGA GGGAAAATTA TCACAATTAC TCATATAATT GAGCCGTCTT TGTAGCAAGT GCAGCTTCAG

                ....|....| ....|....| ....|....| ....|....| ....|....| ....|....| ....|....| ....|....| ....|....| ....|....|
                     605        615        625        635        645        655        665        675        685        695
MmURE2          TAGCCT---T TCTCCATCA- TGAAAATGGT TTCATTATA- GGGTTTTTCA TATTCCCTGA CACCATCTAC -ACAGAGGAG CAAGCGTGCA GATGAGATGT
GgURE2          TAGCCT---T TCTCCATCA- TGAAAATGGT TTCATTATA- GGGTTTTTCA TATTCTCTGA CACCATCTAC -ACAGAGGAA CAGGCGTGCA GATGAGATGT
AcURE2          TAGCCT---T TCTCCATCA- TGAAAATGGT TTCATTATA- GGGTTTTTCA TATTCTCTGA CACCATCTAC -ACAGAGGAA CAGGCGTGCA GATGAGATGT
XtURE2          TAGCCT---T TCTCCTTCA- TGAAAATGGT TTCATTATA- GGGTTTTTCA TATTCCCTGA CACCATCTAC -ACAGAGGAA CAGGCGTGCA GATGAGATGT
CmURE2          TAGCCT---T TTTCCATCA- TGAAACTGCT TTCATTATAT G-TTTTTTCA TATTCTCTGA CACCACGCGC -ACACAGAAG CAGGCGTGCA GATGAGATGC
DrURE2          GAGCCC--TT TTTCCATCAG CCAAAATGGT TTCATTATAG GGG-TTTTCA TATTCCCTGA CACGGGGCGC AGGGGGGGCC GCGGTGTGCG CAGGAGTCGC
OlURE2          TAGCCCCTTT TTTCCATCAA CCAAAATGGT TTCATTATAT GGGTTTTTCA TATTCCCTGA CACAGGGCTC CGCTGAGGGC CTGGCGCGTG GATGAGATGC
TnURE2          TAGCCCC-TT TTTCCATCAG CCAAAATGGT TTCATTATAT GGGTTTTTCA TATTCTCTGA CACAGGGCTC TGCTGAGGGC CTGGCGCGTG GATGAGATGC
TrURE2          TAGCCCCCTT TTTCCATCAG CCAAAATGGT TTCATTATAT GGGTTTTTCA TATTCCCTGA CACAGGGCTC TGCTGAGGGC CTGGCGCGTG GATGAGATGC

                ....|....| ....|....| ....|....| ....|....| ....|....| ....|....| ....|....| ....|....| ....|....| ....|....|
                     705        715        725        735        745        755        765        775        785        795
MmURE2          GCT--GGGAA CAGGCTAGAT CAGTAAGGTC ACAGTAGGAA TAATTAGCTC TGCTATGGAA AGAGCATCCA G-GCCTTTTA CTG------C TACATAAATG
GgURE2          ACT--AGGAA CAGGGTAGAT CAGTAAGGTC ACAGTAGGAA TAATTAGCTC TGCTATGGAA AGAGCATCTA G-GCCTTTTA CTG------C TACATAAATG
AcURE2          ACT--AGGAA TAGGGTAGAT CAGGAAGGTC ACAGTGGGAA TAATTAGCTC TGCTATGGAA AGAGCATCTA G-GCCTTTTA CTG------C TACATAAATG
XtURE2          ACT--CGGAA CAGTGCAGAT CAGCGAGGTC ACAGTAGGAA TAATTAGCTT TGCTATGGAA AGAGCTTGTA G-GCCTTTTA CCG------C TACATAAATG
CmURE2          AGGGGAGGAA CATTACAGAT CAACGAGGTC ACAGTGGGAG TAATTAGCTG TGGTATGGAA AGAGCATCCA G--CCTTTTC TG-------C GGTAGAGATG
DrURE2          TGG--GGGAG AAGGGTGGAT CATTAAGGTC ACAGTGGGGA TAATTATCTT GACTATGGAA AGAGCATCCA GCACCTTTTT TCTCTTCCCC GCCACAAAAG
OlURE2          AGG--ATGAA CAGAGTGAAT CATTAAGGTC ACAGTAGGAA TAATTATCCC AGCTATGGAA AGAGCATCTA AAGCCTTTTT TCTC--CCCC GGCACAAATG
TnURE2          AGG--ATGAA CAGAGTGAAT CATTAAGGTC ACAGTAGGAA TAATTATCCC AGCTATGGAA AGAGCATC-A G--CCTTTAT TCTC--CCCC AGCACAAATG
TrURE2          AGG--ATGAA CAGAGTGAAT CATTAAGGTC ACAGTAGGAA TAATTATCCC AGCTATGGAA AGAGCATC-A G--CCTTTAT TCTC--CCCC AGCACAAATG
```

```
          ....|....| ....|....| ....|....| ....|....| ....|....| ....|....| ....|....| ....|....| ....|....| ....|....|
                 805        815        825        835        845        855        865        875        885        895
MmURE2    TACTGTCCGT GGCTTTTAGC CACAAA--AA AAC-TTACTA ACAAATGGAG CTCCCGCCTA CTACTTTGA- ---------- ------AAAA AAGATTTGTA
GgURE2    TACTGTCCAT GGCTTTTAGT CACAAA--AA AAC-TTACTA ACAAATGGAG CTCCCGCCTA CTACTTTGA- ---------- ------AAAA AAGATTTGTA
AcURE2    TACTGTCCGT GGCTTTTAGT CACACAAAAA AACCTCACTA ACAAATGGAG CTCCCGCCTA CTACTTTGA- ---------- ------AAAA AAGATTTGTA
XtURE2    TACTGTCCGT GGCTTTTAGT CACAGA-AAA AAC-TTACTA ACAAATGGAA CTCCCGCCTA CTACTTTGA- ---------- ------AAAA AAAATTTGTA
CmURE2    TACAATCCAC GGCTTTCAGT CACA---AAG AAC-TTAGTA ACAAATGGAG CTCCCGCCCA TTGCTTTAA- ---------- ------AAAA AAGATTTGTA
DrURE2    CACCGTCCAT GGCCTTTCGG CACA---AAG AAC-TTGGAA ATAAATGGAG CTTCCACCTA CAACTTATTC ACAAA----- ----GAGAGA GAGACTTGTA
OlURE2    CAATGTCCAC GGCTTTTAGT CACA---AAG AAC-TTTGTT ACAAATGGAG CTTCCACCTT CTACTTATAC AAAGAAGAGA GGGGGAGAGC AAGACTTGTA
TnURE2    CAACGTCCAC GGCT------ -------TAG AAC-TTTGTT ACAAATGGAG TTTCCACCTT CCACTTATAC AAAGACGAGA GGGGGAGAGC AAGACTTGTA
TrURE2    CAATGTCCAT GGCT------ -------TAG AAC-TTTGTT ACAAATGGAG TTTCCACCTT CCACTTATAC AAAGACGAGA GGGGGAGAGC AAGACTTGTA

          ....|....| ....|....| ....|....| ....|....| ....|....| ....|....| ....|....| ....|....| ....|....| ....|....|
                 905        915        925        935        945        955        965        975        985        995
MmURE2    TCAACACTAC AATTTTCCAT CATTAAGACT AATAACACAG A-GCCTAGTA TACATCAAGG GGAATAAAAA G-AAAAATCT CACATTCAAG TGGTGG-CTG
GgURE2    TCAACACTAC AATTTTCCAT CATTAAGACT AATAACACAG A-GCCTAGTA TACATCAAGG GGAATAAAAA G-AAAAATCT CACATTCAAG TGGCGG-CTG
AcURE2    TCAACACTAC AATTTTCCAT CATTAAGACT AATAACACAG A-GCCTAGTA TACATCAAGG GGAATAAAAA G-AAAAATCT CACATTCAAG TGGCGGACTG
XtURE2    TCAACACTAC AATTTTCTAT CATTAAGACT AATAACACAG A-GCCTGCTA TACATCAAGA GGAATGAAAA G-AAAAATCT CACATTCAAG TGGCGG-CTG
CmURE2    TCAACACTAC AATTTTCCAT CATTAAGACT AATAATGGAG A-GCTCGGTA TGCATCAAGG GGAACAAAA- --AAATATCT CACATTCAAG TGGCTG-CTA
DrURE2    TCAACATGAC AATTTTCCAT TATTAGGACT AATGGCGGGC T--GCCTTAG GAGATCAAGG GGAG------ ---AGGGGGA CGCGTTCAGG TGT-GC-TGG
OlURE2    TCAACATGAC AATTTTCCAT CATTAAGACT AATGACAGGC ACAGACTGTG GGGATCAAGG GGAGCACAAA A--AAAGAGG CGCATTCAGG TGTTGG-CTG
TnURE2    TCAACATGAC AATTTTCCAT CATTAAGACT AATGACAGGC GCAGACTGTG GGGATCAAGG GGAGCACAAA AA-AGAGAGG TGCATTCAGG TGTTGG-CTG
TrURE2    TCAACATGAC AATTTTCCAT CATTAAGACT AATGACAGGC ACAGACTGTG GGGATCAAGG GGAGCACAAA AAAAGAGAGG CGCATTCAGG TGTTGG-CTG

          ....|....| ....|....| ....|....| ....|....| ....|....| ....|....| ....|....| ....|....| ....|....| ....|..
                1005       1015       1025       1035       1045       1055       1065       1075       1085       1095
MmURE2    GGCGCTGACC TTTGTTCCC- CCTTTTTGTA TACGA----- ---------- ---------- ---CTTAACT CTTT-ACAAA AAAGAGCCAC ACGCCAC
GgURE2    GGTGCTGACC TTTGTTCCC- TTTTTTTGTG TTCAA----- ---------- ---------- ---CTTAACT CTTT-ACAAA AAGGAGCCAC ACGCCAC
AcURE2    GGTGCTGACC TTTGTTCCCC TTTTTTTGTG TACGA----- ---------- ---------- ---CTTAACT CTTT-ACAAA AAGGAGCTAC ACGCCAC
XtURE2    GGTGCTGACC TTTGTTC--- TTTTTTTGTG TATGTGTGTG TGAGTGTGCG CGTGTGTGTG TGTATGCACG ATTTCACAAA AAAGACCCAC ACAGCAT
CmURE2    GGTTCCAACC CTCCTTCCC- --TTTTTGTC TGCGA----- ---------- ---------- ---GTTAACT CTTTACCATA AACCAGCAGA CTGCACA
DrURE2    AGTACTGACC TCTCCCCCGT CT---TTGTT TGGGA----- ---------- ---------- ---CTTAACT CCGA-ACAAA AACCCTA--A AGGGGAT
OlURE2    AGTGCTGACC TCCTTCCTGT CT---TTGTT GGAGA----- ---------- ---------- ---CTTAACT CTTA-ACAAA AAC--TAAAA AGGGT-T
TnURE2    AGTGCTAACC TCCTTCCTGT CT---TTGTT GGAGA----- ---------- ---------- ---CTTAACT CTTA-ACAAA AAC--TAAAA AGGGGGT
TrURE2    AGTGCTGACC TCCTTCCTGT CT---TTGTT GGAGA----- ---------- ---------- ---CTTAACT CTTA-ACAAA AAC--TAAAA AGGGGGT
```

B

	Tetrapods					Teleosts			
	MmURE2	GgURE2	AcURE2	XtURE2	CmURE2	DrURE2	OlURE2	TnURE2	TrURE2
MmURE2	na	0.97	0.96	0.91	0.83	0.70	0.76	0.73	0.75
GgURE2		na	0.98	0.92	0.84	0.70	0.77	0.75	0.77
AcURE2			na	0.91	0.84	0.70	0.76	0.74	0.75
XtURE2				na	0.80	0.66	0.73	0.71	0.73
CmURE2	0.83	0.84	0.84	0.80	na	0.68	0.74	0.71	0.73
DrURE2						na	0.80	0.78	0.81
OlURE2							na	0.93	0.96
TnURE2								na	0.96
