# Supplementary figures and images for: Functional conservation of a forebrain enhancer from the elephant shark (Callorhinchus milii ) in zebrafish and mice
Source: BMC Evol Biol. 2010 May 26;10:157. doi: 10.1186/1471-2148-10-157 (PMC2891724; doi:10.1186/1471-2148-10-157)

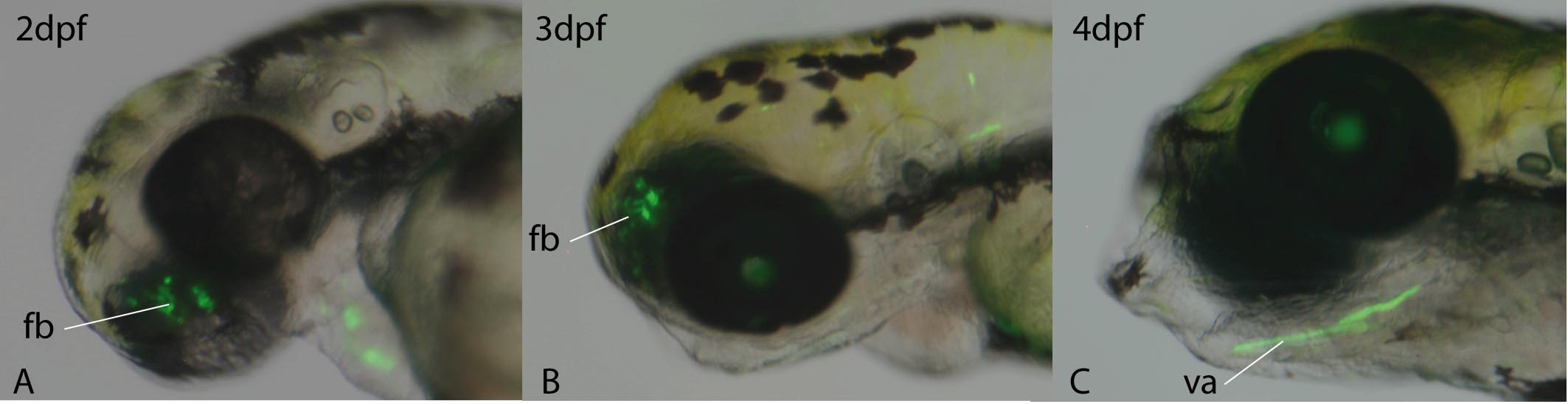

Supplement: Additional file 2 — Primary transgenic zebrafish embryos with gfp expressed under MmURE2 sequence. GFP fluorescence could be detected in the forebrain (fb) of primary transgenic zebrafish at 2 dpf (A) and 3 dpf (B) after injection of the construct. GFP fluorescence was also detected in the visceral arches (va) of 4 dpf old embryos after injection (C). [file 1471-2148-10-157-S2.JPEG]

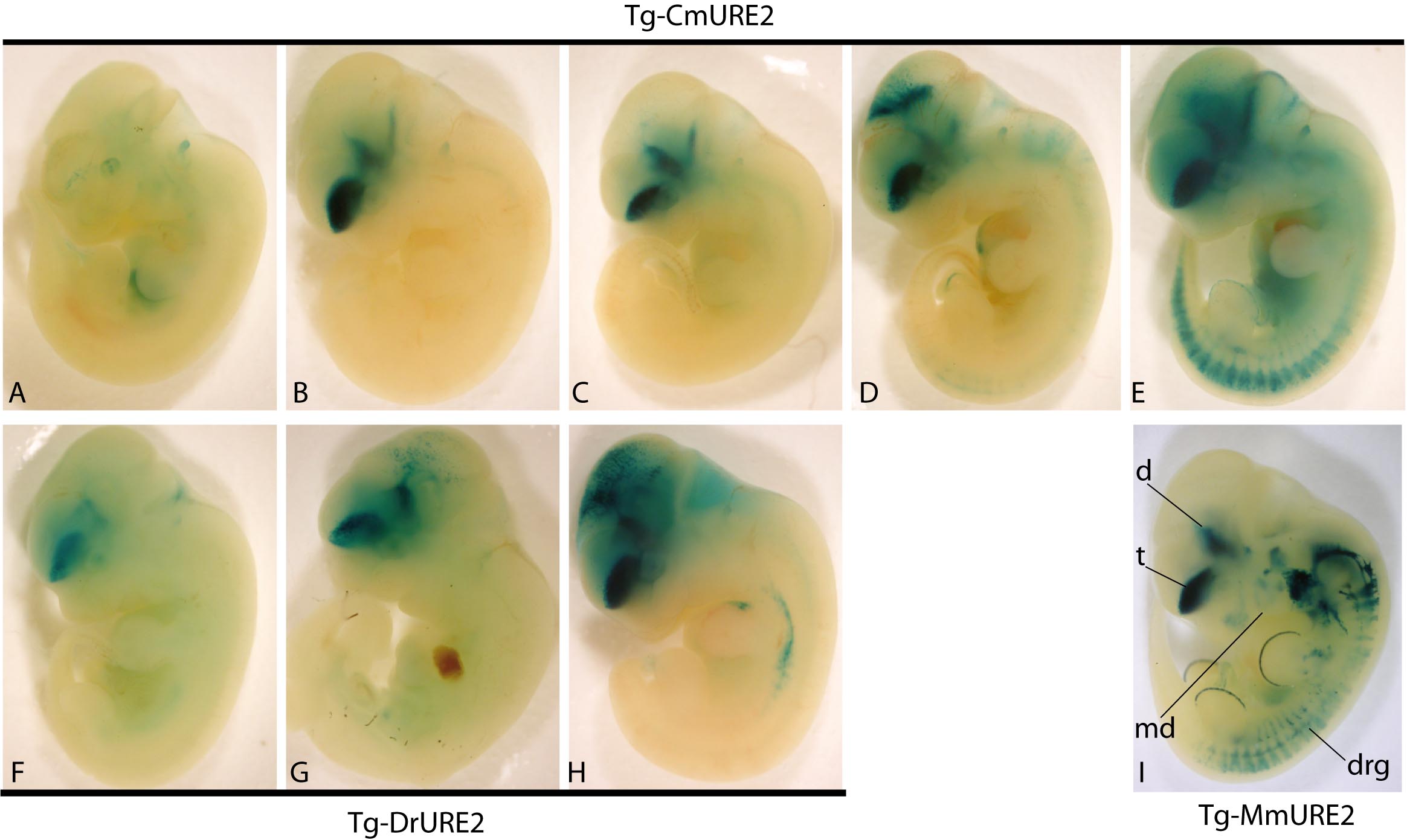

Supplement: Additional file 3 — Primary transgenic mice with LacZ expressed under CmURE2 (A-E), DrURE2 (F-H) sequences to be compared with LacZ expression in a stable transgenic line under MmURE2 regulation (G), at E11.5. d: diencephalon, drg: dorsal root ganglia, md: mandibular arch, t: telencephalon. [file 1471-2148-10-157-S3.JPEG]
